# The Genetic Selection of *HSPD1* and *HSPE1* Reduce Inflammation of Liver and Spleen While Restraining the Growth and Development of Skeletal Muscle in Wuzhishan Pigs

**DOI:** 10.3390/ani14010174

**Published:** 2024-01-04

**Authors:** Yuwei Ren, Feng Wang, Ruiping Sun, Xinli Zheng, Yuanyuan Liu, Yanning Lin, Lingling Hong, Xiaoxian Huang, Zhe Chao

**Affiliations:** 1 Key Laboratory of Tropical Animal Breeding and Disease Research, Institute of Animal Science and Veterinary Medicine, Hainan Academy of Agricultural Sciences, Haikou 571100, China; renyuwei@hnaas.org.cn (Y.R.);; 2 College of Veterinary Medicine, Xinjiang Agricultural University, Urumqi 830052, China

**Keywords:** Wuzhishan pigs, whole genome sequencing, heat shock proteins, inflammation, growth

## Abstract

**Simple Summary:**

The inhibition of inflammation is of great importance for porcine growth and development under heat stress, and due to the hot and humid weather in the Hainan province of China, the local pig populations, including Wuzhishan (WZS) minipigs, have developed excellent heat resistance and adaptability. Moreover, the WZS minipig is a perfect animal model for human disease investigation. Therefore, the genetic selection of genes that play roles in both inhibition of inflammation and suppression of growth is beneficial for the conservation and utilization of WZS minipigs. In this study, we analyzed the genetic selection of genes in top genetically different regions in WZS minipigs and four other pig populations using whole genome sequencing, and we found that the HSP genes (*HSPD1* and *HSPE1*) are connected to both inflammation and growth. As a result, this study offers a valuable theoretical basis for the sustainable application of the conservation and utilization of WZS pigs.

**Abstract:**

Wuzhishan (WZS) pigs, which are minipigs native to Hainan Province in China, are characterized by strong resistance to extreme hot temperatures and humidity. The relationship between their immune response and growth still needs to be clarified. In this study, we used whole genome sequencing (WGS) to detect variations within 37 WZS pigs, 32 Large White (LW) pigs, and 22 Xiangxi black (XXB) pigs, and ~2.49 GB of SNPs were obtained. These data were combined with those of two other pig breeds, and it was found that most of the genes detected (354) were located within the distinct genetic regions between WZS pigs and LW pigs. The network that was constructed using these genes represented a center including 12 hub genes, five of which had structural variations (SVs) within their regulatory regions. Furthermore, RNA-seq and RT-qPCR data for 12 genes were primarily consistent in liver, spleen, and LDM tissues. Notably, the expression of HSPs (*HSPD1* and *HSPE1*) was higher while that of most genes involved in the JAK3-STAT pathway were lower in liver tissue of WZS pigs, compared with LW pigs. This likely not only reduced inflammation-related immune response but also impaired their growth. Our findings demonstrated the role of HSPs in the connection between inflammation and growth rate, while also providing the fundamental genetic selection of the adaptability of WZS pigs.

## 1. Introduction

Pigs were domesticated independently in two clusters simultaneously with the development of agricultural communities and civilizations [1,2], and several populations of breeds with more advantageous traits gradually expanded within diverse geographical regions [3]. One group of pigs was specific in their immune system and were referred to as minipigs [4], such as the Wuzhishan (WZS) pigs from Hainan Province of China. Although they have a small body and a slow growth rate, most of their tissues are similar to those of humans in terms of physiological structure; in addition, the splenic immune functions of pigs and humans share many similarities but show tremendous differences with mice. A study comparing splenic artery and neuromodulation between mice, rats, pigs, and humans indicated that the pig was the most suitable model for investigating human splenic innervation [5]. Göttingen minipigs were implicated in radiation-induced coagulopathy and Alzheimer’s disease recently [6,7]; WZS minipigs were used for an alloxan toxicity test and Islet isolation and purification in a diabetes simulation experiment [8,9]; therefore, minipigs acted as a good model for investigating the human immune system. 

Large-scale gene variation approaches were used to analyze the functionality of the genetic selection achieved in recent years. For example, one study found that immunity-related gene families of Korean native pigs greatly expanded and that specific single nucleotide polymorphisms (SNPs) have been selected during their evolution [10]. Furthermore, many structural variations (SVs) were identified in immune and nervous system genes between Ningxiang and Duroc pigs. An analysis of SNPs of geographically isolated pigs in China revealed specific genetic variations and evolutionary patterns [11]. Also, the WGS analysis of WZS pigs identified several genes associated with immunity and meat quality [12], and the copy number variation (CNV) in wild boars and domestic pigs was used to investigate the association between genes and meat traits, growth, and immunity [13]. Although indigenous pigs commonly exhibit poor growth, they have a great variety of breeds and genetic diversity and are essential for the development of the pig industry; therefore, they deserve investigation for genetic selection and adaptive functions.

Heat shock proteins (HSPs) represent a superfamily of conserved molecules, and many HSPs protect cells from stressful conditions, such as harsh climates and virus infection [14]. HSPD1, also called HSP60, is a double-edged sword in immunity; it is considered as an initiator of oxidative stress and a potential neuroinflammation modulator [15], and it has reduced oxidative injury in skeletal muscle [16,17]. HSPE1, also named HSP10, may cooperate with HSPD1 to ensure protein folding and maintain proteostasis [18]. Moreover, *HSPE1* is an inflammation-related gene because of its increasing expression during inflammatory activation [19]. The JAK-STAT signaling pathway is known to induce inflammation by interacting with several pro-inflammatory cytokines, and many genes are involved in this pathway, including *JAK2*, *JAK3*, *STAT1*, *STAT2*, *STAT3*, and *STAT4*, to regulate multiple functions, namely cellular proliferation, senescence, autophagy, and inflammation activation [20,21].

Since minipigs were introduced and domesticated in Europe in the early 1970s [22], and WZS pigs have also been domesticated for a few decades, simulations from the outside environment would likely stress them, and they were found to be adaptable to wild environments. The ability to maintain a balance between immune response and stress developed through a selective sweep in genome regions during long-term outdoor survival. Heat stress would bring harmful effects to pigs, including early pregnancy disruptions, poor muscle fiber growth, and weight loss [23]. Meanwhile, HSPs are a superfamily of conserved molecules and are known to be important for resistance to stressors, such as extreme temperatures and virus infection [14]; however, which member of the HSPs underwent genetic selection during minipig evolution and how they affect the tradeoff between immune response and growth are not clear yet. In this study, we detected WGS data of 37 WZS pigs, 32 Large White (LW) pigs, and 22 Xiangxi black (XXB) pigs and performed a comparative analysis of these data along with the SNP data for 29 Duroc pigs and 32 Meishan pigs downloaded from the NCBI database to investigate the population structure and genetic selection. Based on the genes within the distinct genetic selection regions between WZS pigs and other pigs, we constructed a core network including HSPs and relevant genes. Moreover, we detected the SVs of WZS and LW pigs to identify the significant variants in genomic regions and genes in the core network, and we used transcriptional levels to analyze the connections of these genes. This study investigated the linkage of HSPs between the immune system and growth rate during the genetic selection process, and it provided favorable information for genetic breeding. 

## 2. Materials and Methods

### 2.1. Samples and Data Sets

The ear tissues of 37 WZS pigs (4-months-old) and 32 LW pigs (2-months-old) were sampled from Hainan and Guangdong provinces separately, and the WZS pigs were domestic ones from the Wuzhishan conservation farm of Chengmaiwhile LW pigs were sampled from a culturing farm of Guangzhou. The ear tissues of 22 XXB pigs (10-months-old) were sampled from a farm in Jishou city of Hunan Province. The ear tissues were preserved in 100% ethanol tubes at −80 °C. The WGS data sets of Duroc (29 individuals) and Meishan (32 individuals) were downloaded from the NCBI database (Accession ID: PRJNA378496) [24]. 

### 2.2. Resequencing and Variant Calling

DNA of the samples was extracted using the DNeasy Blood & Tissue kit (QiaGen, Shanghai, China). DNA integrity was detected on agarose gels, and DNA concentration was quantified using a Qubit 4.0 Fluorometer (ThermoFisher Scientific, 33, Marsiling Industrial Estate Road 3, #7-06, Singapore 739256). At least 1 μg genomic DNA was used to construct paired-end libraries that contained average insert sizes of approximately 350 bp according to the manufacturer’s instructions, and the prepared libraries were inspected using an Agilent2100 Bioanalyzer, quantified using real-time PCR, and then sequenced on the Illumina Nova 6000 platform by the Novogene Corporation (Beijing, China).

The raw data was assessed for quality using FastQC v0.11.9 (https://www.bioinformatics.babraham.ac.uk/projects/fastqc/, accessed on 5 November 2022) and trimmed adapters and low-quality reads by Trimommatic v.0.36 [25]. The filtering high-quality PE150 reads were aligned to the pig reference genome (Sscrofa11.1) using the Burrows–Wheeler aligner (BWA mem) v.0.7.17 (https://sourceforge.net/projects/bio-bwa/files/, accessed on 5 November 2022), and the conversed bam files were sorted using SAMtools [26]. Duplicated reads were filtered using the MarkDuplicates module in GATK v4.2.2.0 (https://gatk.broadinstitute.org/hc/en-us, accessed on 12 December 2022). TheSNPs and indels were called using the HaplotypeCaller module to get a gvcf file for each sample [27], and only sites with a depth >4× were reserved for further study. All the gvcf files were merged using the CombineGVCFs and called SNPs and indels using GenotypeGVCFs, and SNPs were filtered using SelectVariants with the parameter of “–select-type-to-include SNP”, while indels were filtered with the parameter of “–select-type-to-include INDEL”. A VariantFiltering module was performed to filter SNPs with the parameters “QUAL < 30.0|QD < 2.0|MQ < 40.0|FS > 60.0|SOR > 3.0|MQRankSum < −12.5|ReadPosRankSum < −8.0” [28]. The filtered SNPs were annotated using SnpEff according to the known gen structures of reference genome Sscrofa11.1 [29], including exon, intron, intergenic regions, and upstream and downstream regions, as well as synonymous and nonsynonymous mutations. The high-quality SNPs were visualized using TBtools [30].

### 2.3. Population Genetic Differentiation

The high-quality SNPs were used to estimate the genetic diversity and population differences, and the metrics included expected heterozygosity (He), nucleotide diversity (π), Tajima’s D, and FST. Values of π and He were calculated at nonoverlapping 1 Mb distance along the genome of each individual using the vcftools v0.1.17 [31]. The divergences across populations were measured using Weir and Cockerham’s estimator of FST [32] using the vcftools v0.1.17 with 300 Kb bins. Linkage disequilibrium (LD) decay reflected non-random associations between alleles at different loci within a population, which was evaluated using the parameter r^2^ with a maximum distance of 300 kb using PopLDdecay v3.41 [33].

### 2.4. Population Structure

The identity-by-state (IBS) genetic distance matrix between the individuals was calculated using PLINK v1.90 and visualized using a phylogenetic tree performed with a neighbor-joining (NJ) algorithm through the SplitsTree5 v5.1.7-beta [34]. PCA was analyzed using the R package to show the clustering of samples. Moreover, model-based clustering was constructed utilizing the admixture (http://dalexander.github.io/admixture/, accessed on 5 March 2023) with the number of clusters K from 2 to 8. The optimal K was determined via the minimal value of Coefficient of Variation (CV) error.

### 2.5. History Demographic Estimation

The PSMC model [35] was used to reproduce historically effective population size (Ne) changes through the evolutionary process. A mutation rate of 3.6 × 10^−9^ mutations per nucleotide site per generation and a generation time of three years were set as scale labels into years and individuals for calculating N_e_ [36]. The result was visualized from 1 × 1000 years ago (Kya) to 1000 Kya.

### 2.6. Structural Variation Calling

SVs were called from the sequences (>15×) of three WZS pigs and three LW pigs (4-month-old sows, with weight values in Supplementary Table S1) and mapped to the reference genome. Sniffles and PBSV with default parameters were used to detect SVs including deletions (DELs), inversions (INVs), and duplications (DUPs) with a size range of 20 bp~1 Mb. SVs of three replicated samples of two breeds were combined separately to generate the common SVs of each breed, and then the different SVs in genes between the two breeds were then compared to select the specific SVs for WZS pigs. 

### 2.7. RNA-Seq Analysis

The pigs were purchased from the Wuzhishan preservation factory (Chengmai, Hainan), and these pigs were maintained in a similar environment with free food and water for an adaptive week before slaughtering. After the transitional period, all the pigs had their feed withdrawn for 24 h, and they were transferred to the slaughterhouse of the HAAS Yongfa pig experimental facility. The pigs were stunned via electric shock and exsanguinated, flushed, and split without consciousness. The liver, spleen, and Longissimus Dorsi muscle (LDM) tissues were collected from three WZS pigs and three LW pigs (4-month-old sows, with weight values in Supplementary Table S1, and samples the same as those in Section 2.6). Samples were collected from the middle part of the right lateral lobe of porcine liver, and the middle part of spleen and Longissimus Dorsi muscle. All the samples were frozen directly in liquid nitrogen and transferred to the library and stored at −80 °C before RNA extraction. RNA was extracted from tissues using an RNA extraction kit (Qiagen, Cat. No./ID: 74104) to perform RNA-seq on the Illumina Nova 6000 platform by the Novogene Corporation (Beijing, China), and each sample obtained ~6 GB data. Raw reads of RNA-seq were filtered using Trimmomatic (Version 0.36.6) to filter adapters and low-quality reads, clean reads were mapped to the reference genome (Sscrofa11.1) using HISAT2 with default parameters (Version 2.2.1) [37], and read counts were calculated using HTSeq v. 0.9.1 [38]. Differentially expressed genes (DEGs) were identified using DEseq2 [39] according to the requirements of |log_2_ (foldchange)| ≥ 1, and they were adjusted by *p* ≤ 0.05.

### 2.8. Functional Enrichment

The pathways and functional classification of genes in the top 5% distinct genetic selection regions and DEGs were arranged using the R package clusterProfiler [40] based on KEGG [41], and the results were statistically adjusted by *p* ≤ 0.05. Protein–protein interaction (PPI) networks were constructed using genes corresponding to the top 5% distinct genetic selection regions between WZS and LW pigs using STRING (https://cn.string-db.org, accessed on 12 April 2023). Subsequently, the visualization of the enrichment results was implemented using R software (R 4.2.3). Furthermore, the domains of the selected genes from the PPIs were analyzed using the Pfam database (http://pfam-legacy.xfam.org/, accessed on 7 May 2023).

### 2.9. RT-qPCR

The hub genes were selected from core PPIs in the Section 2.8, and expression levels were identified using RT-qPCR. The cDNA samples were the same as in the Section 2.7, and the primers are referred to in Supplementary Table S2. The beta-actin was used as reference gene for liver and spleen tissues, and GAPDH was the reference gene for LDM. The gene expression was calculated and presented as 2^−ΔΔCT^ values, the statistical values were confirmed via Student *t*-test, and gene expressions with *p* value ≤ 0.05 were significantly changed in the tissues between WZS and LW pigs. 

## 3. Results

### 3.1. Genomic Variant Map

A total of 91 samples collected from three pig breeds (WZS, LW, and XXB) were sequenced using WGS, and more than 2661 Gb of raw data were obtained, with an average depth of 11.56× per sample and average coverage of 92.42% based on the the Sscrofa11.1 genome sequence downloaded from the NCBI database (Supplementary Table S3). After trimming, approximately 99.27% of the clean data (more than 2641 Gb) remained (1012, 879, and 750 Gb for WZS, LW, and XXB, respectively). The clean data were mapped to the reference genome to detect variants, resulting in a total of ~2.49 Gb of SNPs: ~35.55, ~17.1, and ~28.23 Mb of SNPs were found in WZS, LW, and XXB genomes, respectively. Furthermore, SNPs were also called from data downloaded for Duroc and Meishan pigs and used for the genetic analysis together with SNPs called for the other three populations. 

**Figure 1:**
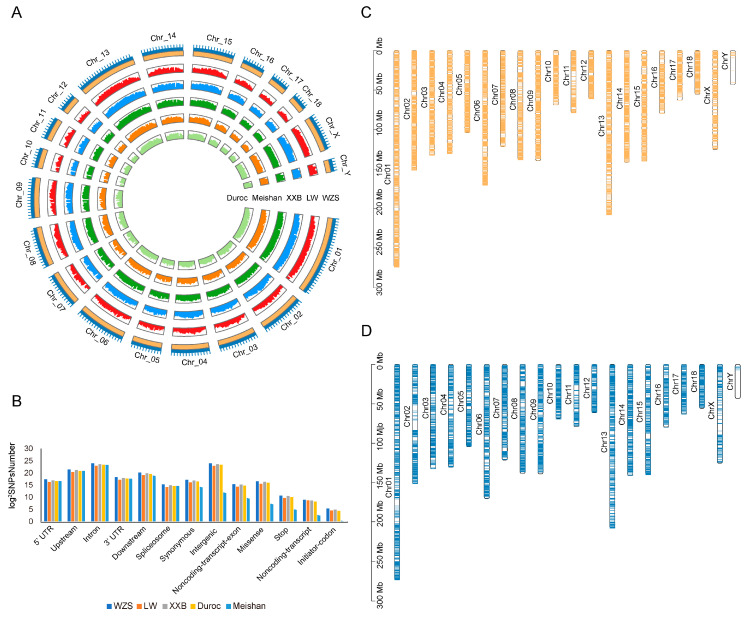
Map of genomic variants for five pig breeds. (**A**) Density of SNPs on 20 chromosomes for five pig breeds. Light green, light orange, dark green, blue, and dark orange represented Duroc, Meishan, XXB, LW, and WZS pigs, respectively. The outermost layer represented 20 chromosomes. (**B**) Structural annotation of five pig breeds. (**C**,**D**) The distribution of SVs on 20 chromosomes of WZS pigs (**C**) and LW pigs (**D**).

The average WZS SNP densities were 1473.12/kb, 912.54/kb, and 238.16/kb for the autosomes, X chromosome, and Y chromosome, respectively (Figure 1A). The SNPs were randomly distributed in the genome, with approximately 8.29% in upstream regions, 3.40% in the downstream regions, 47.04% in the intron, and 1.37% in UTRs, showing that a large proportion of SNPs were located in non-coding and regulatory regions; in addition, 0.43% of the SNPs were synonymous variants (Figure 1B), 331 SNPs were located at a start codon, and 1798 SNPs were located at a stop codon (Supplementary Table S4). 

SV calling revealed 81,545 SVs (41,135 deletions, 40,043 insertions, 92 duplications, and 275 inversions) across WZS pigs and 43,647 SVs in LW pigs (20,776 deletions, 22,756 insertions, 28 duplications, and 87 inversions). Moreover, more SVs were located in genes in WZS pigs (10,632 genes) than in LW pigs (6999 genes) (Figure 1C,D).

### 3.2. Genetic Relationship and Population Structure

**Figure 2:**
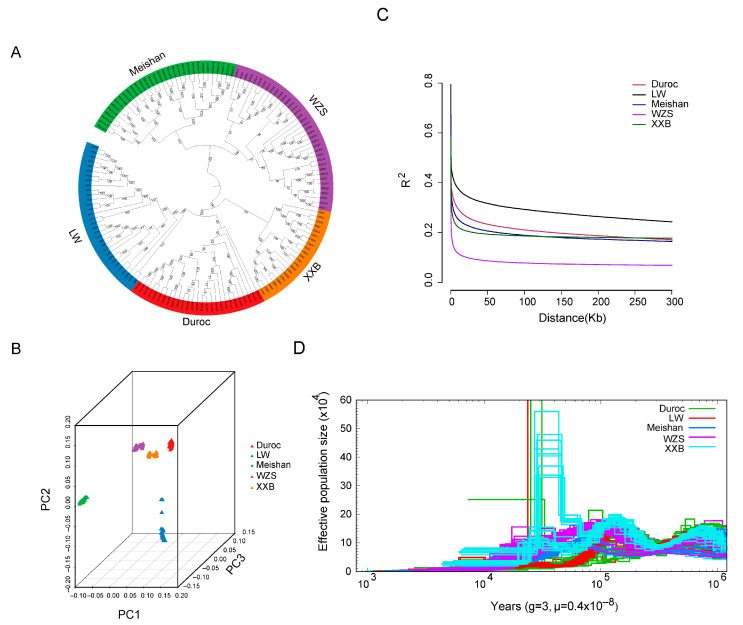
The population structure of five pig breeds. (**A**) A phylogenetic tree of five pig breeds generated using the neighbor-joining algorithm. (**B**) Data collected from the PCA analysis. (**C**) The linkage disequilibrium value. (**D**) The change in total effective population size for five pig breeds.

The phylogenetic relationship was assessed using the whole genomic SNPs of five pig breeds with those of 152 unrelated individuals (37 WZS pigs, 32 LW pigs, 22 XXB pigs, 32 Meishan pigs, and 29 Duroc pigs). The five pig breeds were present in five different clades in the phylogenetic tree (Figure 2A), which was consistent with their different geographical distributions. Within the phylogenetic tree, WZS pigs and Meishan pigs were genetically closer to each other, followed by XXB pigs, while they were mostly distantly related to LW pigs. In addition, Duroc and LW pigs were separated from three indigenous pig breeds, delineating the distance between foreign and indigenous pigs. 

The geographic distances were further supported by principal component analysis (PCA) and population structure based on an Admixture model [42]. In detail, the PCA plot differentiated the 152 individuals into five geographic groups (Figure 2B). Additionally, the smallest cross-validation error was observed at the optimal number K = 7 (Supplementary Figure S1), with four indigenous and two foreign pig blocks of five pig populations divided into six genetic clusters, two of which are represented by WZS individuals separated from other breeds. Linkage disequilibrium (LD) analysis indicated that the half-maximal value of the physical distance between SNPs was ~158 kb (r^2^ = 0.073) for WZS pigs (Figure 2C), which was the lowest coefficient at the same distance among five populations, indicating that WZS pigs had the shortest domestication time. 

### 3.3. Effective Population Size

Pairwise sequentially Markovian coalescent (PSMC) analysis demonstrated that the effective population size (Ne) of the five pig populations was small and changed slowly more than 10 thousand years ago (Kya); however, XXB pigs experienced a dramatic expansion in population size at approximately 10–100 Kya, with the Ne reaching more than 60 × 10^4^. Simultaneously, a small part of LW and Duroc pigs showed an expansion followed by a population reduction, while WZS and Meishan pigs have showed a small Ne with stable fluctuation during the entire evolutionary period (Figure 2D). Moreover, WZS and XXB pigs could be classified into two clusters in relation to history differentiation approximately at 100 Kya and at 60 Kya, separately; Meishan pigs might experience divergence before 1000 Kya as the individuals were dispersive during 1000 Kya; Duroc and LW pigs did not display apparent divergence (Figure 2A,D). 

### 3.4. Genetic Diversity

**Figure 3:**
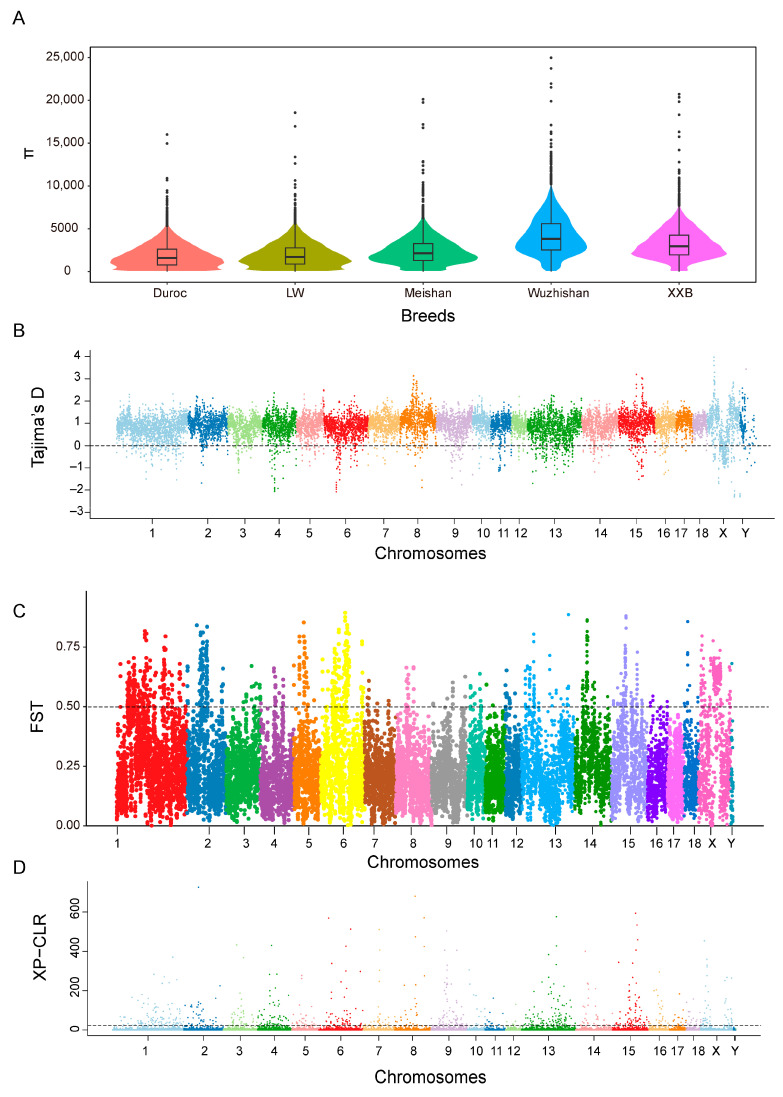
Differences in variation between WZS pigs and other pigs. (**A**) The π value of five pig breeds. (**B**) The Tajima’s D value of WZS pigs. (**C**) The FST value between WZS and LW pigs. (**D**) The XP-CLR value between WZS and LW pigs.

The nucleotide diversities (π) of the WZS, LW, XXB, Duroc, and Meishan pig populations were estimated using a bin size of 300 kb, and the highest average π value (0.004) was obtained for WZS pigs and the lowest π values (0.001) for LW and Duroc pigs (Figure 3A). Additionally, both the expected heterozygosity (HE) and observed heterozygosity (HO) of WZS pigs were larger than those of Duroc pigs and LW pigs, indicating the relatively low heterozygosity of WZS pigs compared with other domestic pigs. Furthermore, most of the Tajima’s D values were not equal to zero for five populations (Figure 3B and Figure S2), indicating that all the populations were subjected to artificial selection. 

The FST value between WZS pigs and Meishan pigs (FST = 0.176) was less than that between the other two breeds, suggesting that the WZS and Meishan pig populations share a closer genetic relationship. In contrast, the high FST value between WZS and Duroc pigs indicates the substantial divergence of these populations, similar to that between WZS and LW pigs (Figure 3C and Figure S3). Next, we calculated the XP-CLR value, which measures the degree of selective sweep, to identify regions under selection during short-term artificial selection or long-term natural selection, which may be necessary for adaptation. The XP-CLR detection results were all selected with high values on genetic selection regions between WZS and LW pigs (Figure 3D and Figure S4), gradually setting the genotypes of this area.

### 3.5. Functional Annotation of the Most 5% Distant Genes

**Figure 4:**
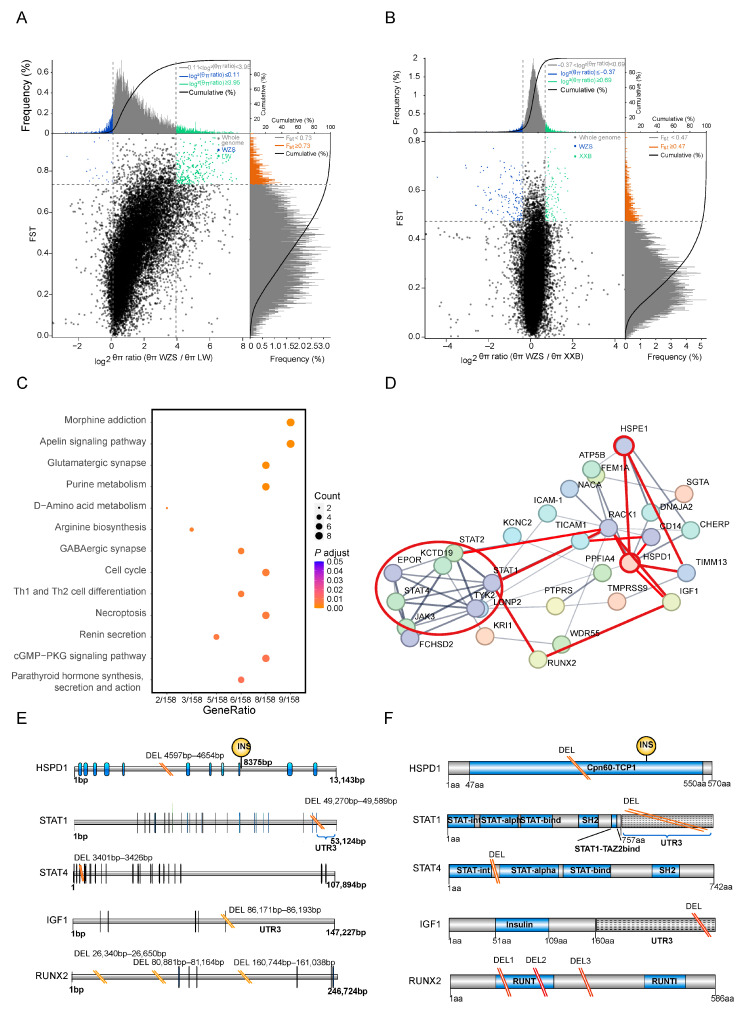
SNPs and genes corresponding to the top 5% genetically different regions selected from WZS and LW pigs selected from WZS and LW pigs. (**A**,**B**) The SNPs in the top 5% genetically different regions to be calculated of FST values and θ_π_ ratios between (**A**) WZS and LW pigs, (**B**) and between WZS and XXB pigs. (**C**) The KEGG enrichment analysis of the genes corresponding to the top 5% genetically different regions between WZS and LW pigs. (**D**) PPI network of the genes corresponding to the top 5% genetically different regions between WZS pigs and LW pigs. (**E**) The SVs in the CDS of five genes detected in WZS pigs. (**F**) SVs in the functional domains of five genes detected in WZS pigs.

The top 5% genetically different regions were those including the regions with top 5% FST values and top 5% θ_π_ ratios among the population pairs to determine the artificially selected windows. The maximum number of genes were identified within the top 5% distinct genetic regions between WZS and LW pigs (354 genes) (Figure 4A), followed by WZS and XXB pigs (216 genes) (Figure 4B; Supplementary Table S5). 

KEGG pathways enrichment analysis was performed for genes corresponding to the top 5% genetically different regions selected between WZS pigs and other populations. The differences between WZS and LW pigs were mainly in the nervous system pathway and immunity containing Th1 and Th2 cell differentiation and necroptosis (Figure 4C; Supplementary Table S6). By contrast, the enrichment analysis of genes with high diversity scores between WZS and XXB pigs revealed differences in the oxytocin signaling pathway, fatty acid metabolism, and arginine biosynthesis (Supplementary Table S7; Supplementary Figure S5B). In addition, the prolactin signaling pathway was enriched for genes with high diversity between WZS and Meishan pigs (Supplementary Figure S5A), indicating a relevant diversity regarding reproductive capacity between WZS pigs and other breeds. As a result, the functions of genes in the top 5% genetically different regions could be divided into three aspects, including immune response, nutrition metabolism, and reproductive capacity, and the immune response and growth and development related to WZS and LW pigs will be selective analysis in this study.

Next, protein–protein interaction (PPI) networks were constructed using genes corresponding to the top 5% of genetically different regions selected from WZS and LW pigs. There were strong connections between several hub proteins. A total of 12 genes formed the main hub of the PPI network of inflammation, skeletal muscle growth, and development, which were linked by HSPD1 and HSPE1 (Figure 4D). The JAK3-STAT pathway proteins JAK3, STAT1, STAT2, STAT4, and TICAM1, located in the same cluster (red circle in Figure 4D), were connected to HSPD1 and HSPE1 either directly or indirectly by other genes. Furthermore, HSPs were linked to IGF1 and RUNX2, two proteins critical for the growth and differentiation of skeletal muscle linked to STAT1. Therefore, the association of inflammation and cellular growth with HSPs formed a triangle at the center of the PPI network. Moreover, other proteins associated with immune response are marked with red lines, such as TIMM13 correlated with an oxidative injury, RACK1, which is involved in the immunity and metabolism, and CD14, a marker of monocytes for the phagocytosis.

### 3.6. SV Detection in Genes in the PPI Network

Interestingly, SVs were identified in regulatory regions of five PPI hub genes in WZS pigs, but not in LW pigs (Supplementary Table S8). The *HSPD1* gene had a 58 bp intronic deletion between coding sequences (CDS) 5 and CDS6. Since the Cpn60-TCP1 domain of HSPD1 was encoded by CDS1 to CDS11, the deletion may affect the function of this domain, similar to the insertion between CDS9 and CDS10 (Figure 4E). *STAT1* and *STAT4*, which encode proteins with similar domains, had similar deletion SVs: a 320 bp deletion located in the UTR3 region of *STAT1* and a 26 bp deletion located between the STAT-int and STAT-alpha domains. The growth hormone-related gene *IGF1* had a 23 bp TATA-box deletion in the UTR3 region, and *RUNX2* had two intronic deletions between the exons encoding the RUNT domain (Figure 4F). However, the gene structure of *HSPE1* was relatively conserved in WZS and LW pigs, and SVs were not detected in this study.

### 3.7. Functional Analysis of Differentially Expressed Genes between WZS and LW Pigs

**Figure 5:**
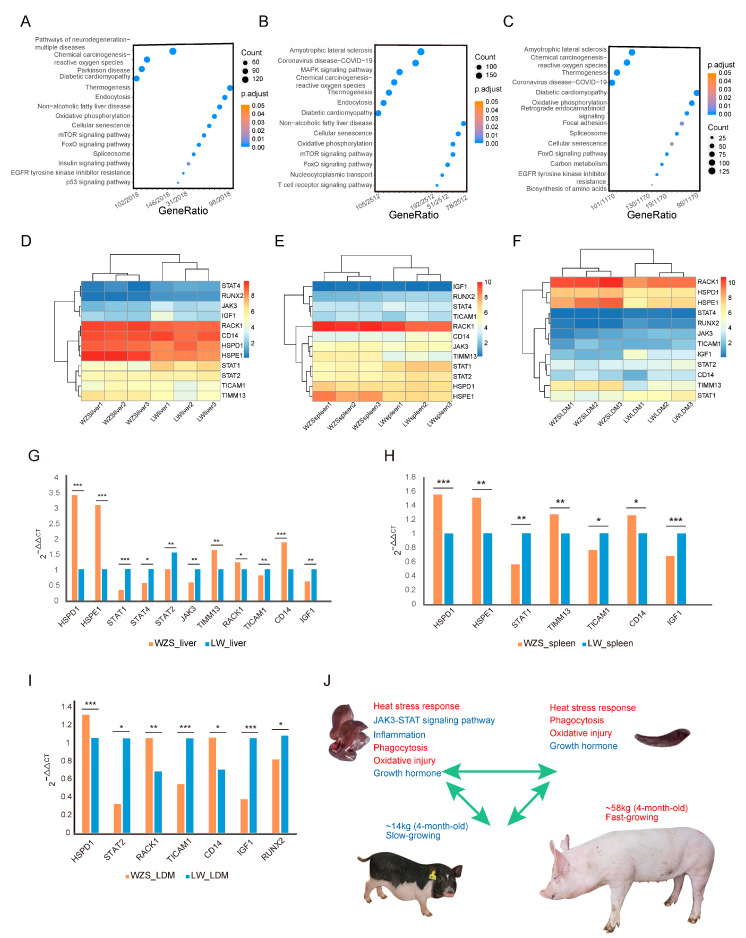
The KEGG enrichment analysis of DEGs in different tissues between WZS and LW pigs. (**A**–**C**) KEGG enrichment analysis results for (**A**) liver tissue, (**B**) spleen tissue, (**C**) and LDM tissue. (**D**–**F**) The expression of 12 genes encoding proteins in the PPI network in (**D**) liver tissue, (**E**) spleen tissue, and (**F**) LDM tissue. The RT-qPCR results for 12 genes in different tissues of WZS and LW pigs: (**G**) liver tissue, (**H**) spleen tissue, (**I**) and LDM tissue. The symbols “*”, “**”, “***” represented 0.01 ≤ *p* value ≤ 0.05, 0.001 ≤ *p* value ≤ 0.01, *p* value ≤ 0.001, respectively. (**J**) Linkage model of immune response and growth state. The figure at the top left corner represents the liver tissue, and the top right represents the spleen tissue of WZS pigs. The pigs at the bottom are 4-month-old WZS and LW pigs.

As the largest number of genes located in regions with high diversity scores were observed between WZS and LW pigs, the differentially expressed genes (DEGs) between the two breeds in different tissues were analyzed. A total of 4626 DEGs, 6129 DEGs, and 2598 DEGs were found in liver, spleen, and LDM tissues, respectively, with the DEGs in the liver mainly enriched in non-alcoholic fatty liver disease, endocytosis, and the p53 signaling pathway, indicating a difference in fatty metabolism and immune response between WZS and LW pigs (Figure 5A; Supplementary Table S9). DEGs in the spleen, which is one of the primary immune organs, were mainly enriched in the immunity of reactive oxygen species (ROS), endocytosis, and T-cell receptors (Figure 5B; Supplementary Table S10). The main enriched pathways for DEGs in the LDM were carbon and lipid metabolism and oxidative phosphorylation (Figure 5C; Supplementary Table S11). 

The expression levels of the 12 hub protein-encoding genes from the PPI network were obtained using RNA-seq data (Supplementary Table S12). *HSPE1*, *TIMM13,* and *RACK1* were more highly expressed in livers of WZS pigs than in the livers of LW pigs. By contrast, *JAK3*, *STAT1*, *STAT4*, and *IGF1* were expressed at significantly lower levels (Figure 5D). Similarly, in the spleen, the expression of *HSPE1* and *TIMM13* was significantly higher, while that of *STAT1* and *STAT2* was significantly lower (Figure 5E). In the LDM, *HSPD1* and *HSPE1* were significantly upregulated in WZS pigs along with *TIMM13* and *RACK1*. On the contrary, *JAK3* and *STAT1* were downregulated, along with the growth-related genes *IGF1* and *RUNX2*, which showed significantly lower expression in WZS pigs than in LW pigs (Figure 5F). 

The expression levels of the 12 hub protein coding genes were also investigated using RT-qPCR (Supplementary Table S2), and most genes had similar expression trends to most genes determined from RNA-seq data (Supplementary Table S13). In detail, *HSPD1*, *HSPE1*, and *CD14* were significantly upregulated, while genes in the JAK3-STAT signaling pathway (*JAK3*, *STAT1*, *STAT2*, *STAT4*, and *TICAM1*) were significantly downregulated in the liver of WZS pigs when compared to LW pigs (Figure 5G). The expression of HSPs was also significantly higher in the spleen, whereas *STAT1* and *TICAM1* expression was significantly lower (Figure 5H). The growth hormone-related gene *IGF1* was significantly downregulated in three tissues, and the skeletal muscle development genes were significantly downregulated in the LDM of WZS pigs compared with LW pigs (Figure 5I), indicating an association between HSPs and inflammation and skeletal muscle growth. Since the most inflammatory-related DEGs were identified in liver tissue, we speculated that HSPs protect the liver by inhibiting the JAK3-STAT pathways; however, skeletal muscle growth was impaired as indicated by the extremely reduced weight of 4-month-old WZS pigs compared to that of LW pigs (Figure 5J). Importantly, other immune responses, including heat stress signaling, phagocytosis, and oxidative damage stress, were enhanced, along with a reduction in inflammation suggesting a relevant role in the resolution of the inflammation reaction.

## 4. Discussion

In this study, WGS was used to analyze the population structure, and the adaptive selection patterns during the evolutionary process of pigs [43]. In the phylogenetic tree analysis, the genetic relationship was the most distant between WZS and LW pigs, and two foreign pig breeds were separated from three indigenous pig breeds. In contrast, XXB pigs acted as a bridge to connect WZS pigs and Duroc pigs, which might be due to the fact that XXB pigs owned both the features of indigenous pigs and foreign pigs, including the immune response ability of WZS pigs and growth rate and body size of Duroc pigs. The divergent genes were then selected by combining the genomic regions with the top 5% FST values and 5% θ_π_ ratios between WZS pigs and other breeds, and the most genes were annotated in the distinct genetic selection regions between WZS and LW pigs, followed by those between WZS and XXB pigs. A majority of Chinese black pigs exhibit similar characteristics. For example, Beijing black pigs are featured with desirable body shape, tender meat quality, and robust disease resistance and are genetically closer to LW and Duroc pigs [44]; Dahe black pigs have good fattening performance, fast weight gain, and high consumer acceptance [45]; Wannan black pigs presented advantages of disease-resistant, high fertility, and a crude-feed tolerance [46]. It is probable that XXB pigs also share these characteristic and present similar immune ability with WZS while having differences in reproductive traits when compared to WZS pigs. The PPI network of the top 5% divergent genes between WZS and LW pigs demonstrated that their functions were connected to JAK3-STAT signaling pathway-mediated inflammation, HSPs that regulated heat stress response, and *IGF1* and *RUNX2*, which are known to be related to growth hormone response and muscular development. Notably, most of the genes in the PPI network hub were connected to HSPs. SVs of three replications from WZS and LW pigs were also detected, and a total of five hub PPI network genes showed SVs in WZS pigs, but not in LW pigs. In detail, the SVs were located within the UTR3 regions of *STAT1* and *IGF1*, and in the introns between exons of *HSPD1*, *STAT4*, and *RUNX2*, which might affect the functional domains encoded by these genes. Consistent with these genes being under genetic selection, RNA-seq and RT-qPCR confirmed that a higher expression of HSPs could have protective effects on liver tissue by inhibiting inflammation mediated by the JAK3-STAT signaling pathway; however, this higher expression also reduced skeletal muscle growth.

HSPs are highly conserved and widely expressed in different tissues; HSPs can interact to form co-chaperonin [47]. There are controversial opinions about the roles of these two genes in immune functions. Indeed, it was reported that the HSPD1-derived altered peptide ligand reduced the pro-inflammatory cytokines in rheumatoid arthritis disease and also in COVID-19 patients [48]; in addition, HSPE1 inhibited lipopolysaccharide-induced inflammation by interacting with HSPD1 [49]. However, HSPD1 was also found to be upregulated and to act as an initiator of oxidative stress and neuroinflammation in diabetes [15], and HSPE1 stimulated mitochondrial stress and damaged the mitochondrial structure of chondrocytes [50]. In this study, although both *HSPD1* and *HSPE1* were significantly more highly expressed in the liver and spleen tissues of WZS pigs compared with LW pigs, the expression of most JAK3-STAT pathway-related genes (*JAK3*, *STAT1*, *STAT2*, *STAT4*, and *TICAM1*) was significantly lower in liver tissue. Meanwhile, the expression of *STAT1* and *TICAM1* was significantly reduced in spleen tissue. The HSPD1 is associated with enhanced phosphorylated protein levels of JAK/STAT (STAT1 and STAT3) signaling pathway proteins, leading to inflammation [16]. Many viruses, such as swine fever virus and porcine reproductive and respiratory virus, have activated the JAK3-STAT pathway to release cytokines. Although moderate levels of cytokines could stimulate host immune responses for protection, myriad cytokines destroy cell homeostasis and cause damage [51,52]; therefore, the significantly reduced gene expressions in the JAK3-STAT pathway might be a developed protective mechanism for WZS pigs. On the other hand, the upregulated expression of HSPD1 contributed to increased cell viability while reducing apoptosis and oxidative damage in H_2_O_2_-treated cells [17]. Skeletal muscle produced several HSPs, including HSPD1 during exercise, and, notably, higher basal HSPD1 protein levels were detected in muscles from endurance athletes than in those of sedentary subjects [53], indicating the role of HSPD1 in maintaining skeletal muscle homeostasis by reducing oxidative stress both during exercise and under pathological conditions [54].

Additionally, heat stress not only increased the transcriptional level and protein level of HSP70, but also caused splenic inflammation and antioxidant suppression, while diary hydroxy-selenomethionine could reduce splenic oxidative damage, apoptosis, and inflammation by suppressing the expressions of STAT1 and STAT3 in pigs [55], which proved the associations between heat stress, HSP70, STAT signaling, and inflammation. Similarly, the proteins encoded by *HSPD1*, *HSPE1*, and JAK3-STAT pathway-related genes strongly interacted with each other within the PPI network, suggesting that HSPD1 and HSPE1 could protect the liver tissue by inhibiting JAK3-STAT-induced inflammation in WZS pigs. Furthermore, HSP expression levels were upregulated in the spleen whereas *STAT1* was downregulated, indicating that the effects of heat stress were similar among tissues. *TIMM13* and *CD14* gene expressions were also significantly higher in the liver and spleen. *TIMM13* upregulation causes reactive oxygen species (ROS) production, oxidative injury, and lipid peroxidation of mitochondria [56], and *CD14* regulates monocyte differentiation and macrophage phagocytosis [57]. Thus, there was more oxidative injury and phagocytosis during the immune response in WZS pigs than in LW pigs.

Heat stress-induced negative effects on porcine growth rates were accompanied by increased gene expressions of HSPs (*Hsp90*, *Hsp70*, and *Hsp25/27*) in muscle satellite cells [58]. Moreover, the weight gain and feed intake of pigs under heat stress treatment were lower than those of pigs housed in normal temperature conditions, while the transcriptional expression level of *Hsp90* in longissimus was higher for the former pigs [59]. The expressions of *HspB6* and *HspA6* genes significantly increased in the porcine jejunum when treated with heat stress, and an important role for *HspB6* was regulating smooth muscle contraction [60]. On the contrary, supplementation with dietary oregano essential oil (OEO) and vitamin E was able to relieve the stress-induced impairment by significantly decreasing the gene expressions of *Hsp27* and *Hsp70* in the muscle, while enhancing meat quality by significantly increasing porky pH or decreasing drip loss values compared to pigs without any supplementations [61]. Therefore, heat stress, HSP expressions, and meat quality are believed to be connected. In this study, according to the PPI network, proteins HSPD1 and HSPE1 also interacted with IGF1 and RUNX2, which are associated with the growth and development of skeletal muscle cells and osteoclasts. IGF1 combined with growth hormone has several effects on different organs [62], one of which is the facilitation of the growth and development of muscle fibers. *RUNX2* induces osteoblast differentiation by regulating the expression of bone matrix protein genes to stimulate osteoblast maturation [63]. Moreover, *RUNX2* was a hub gene connected with *STAT1* through other genes in an analysis of host and gut microbial communities in Duroc pigs [64]. Similar to a previous study, *RUNX2* and *STAT1* indirectly interacted with each other; however, the growth- and development-related genes *IGF1* and *RUNX2* showed significantly lower expression in WZS pigs than in LW pigs, which is consistent with the smaller body size of WZS pigs.

WZS pigs have developed an excellent strategy to adapt to the hot and humid weather in the Hainan province of China [12]. Heat stress is harmful to their health while also destroying the immune system and metabolic balance of their body. Therefore, the increasing HSP expression may act as a warning sensor for their immune system, followed by ROS-induced oxidative injury, further enhancing phagocytosis and suppression of inflammation and immune microenvironment balance. However, growth hormone signaling, and skeletal development are somewhat inhibited, potentially because the priority is to maintain immune response balance while sacrificing growth. Nevertheless, the strong connections between heat stress, inflammation, growth hormone responses, and the underlying mechanism deserve further study. Indeed, based on the experiments and analysis performed in this study, increasing HSP expressions could play a crucial role in suppressing inflammation and protecting WZS pigs by reducing the expression of JAK3-STAT signaling pathway-related genes. Although the associations between the two HSPs (*HSPD1* and *HSPE1*) and most JAK3-STAT pathway-related genes were identified, the regulatory pattern inside the HSPs, immune response, and growth-related genes are unclear, so they still need further investigation via more molecular biology experiments. 

## 5. Conclusions

In conclusion, WZS and Meishan pigs were more genetically close to each other, while WZS and LW pigs presented substantial genetic differences. Moreover, the most genes were annotated in the distinct genetic selection regions between WZS and LW pigs, and a total of 12 hub genes were obtained from the PPI network, which was significantly different in the transcriptional level expressions in the liver, spleen, and LDM tissues between two breeds. The increasing expressions of *HSPD1* and *HSPE1* were associated with the decreasing expression of most JAK3-STAT signaling pathway-related genes in the liver and spleen, and they were also connected to *IGF1* and *RUNX2* genes in LDM, which played a crucial role in the protection pattern while restraining the muscle growth and development for LDM. This study provided relevant insights into the importance of genetic selection of inflammation- and growth-related genes for the ability of WZS pigs to adapt to their environment and contributed to genetic breeding for WZS pigs.

## Data Availability

Raw sequencing data included in this study has been made available in the National Center for Biotechnology Information with the accession numbers of PRJNA994680 and PRJNA1030798.

## Supplementary Materials

The following supporting information can be downloaded at: https://www.mdpi.com/article/10.3390/ani14010174/s1. Figure S1. The optimal number K = 7 of the population structure; Figure S2. The Tajima’s D distribution on the chromosomes of four pig populations. (A) LW. (B) XXB. (C) Meishan. (D) Duroc; Figure S3. The FST distribution on the chromosomes between WZS pigs and other pigs. (A) WZS and XXB. (B) WZS and Meishan. (C) WZS and Duroc; Figure S4. The XP-CLR distribution on the chromosomes between WZS pigs and other pigs. (A) WZS and XXB. (B) WZS and Meishan. (C) WZS and Duroc; Figure S5. The KEGG enrichment of the top 5% genetic area between WZS and other pigs. (A) WZS and Meishan. (B) WZS and Duroc; Table S1: The weight values of pigs for structural variations detection; Table S2: The primers of RT-qPCR for 12 genes; Table S3: Summary information of different breeds of pigs in this study; Table S4: The structure location of SNPs of 5 pig breeds; Table S5: The genes of top 5% genetic selection region between WZS pigs and other pigs; Table S6: The KEGG enrichment of the top 5% between WZS pigs and LW pigs; Table S7: The KEGG enrichment of the top 5% between WZS pigs and XXB pigs; Table S8: The SVs of 5 genes from PPI network; Table S9: The KEGG enrichment of the DEGs from liver tissue between WZS pigs and LW pigs; Table S10: The KEGG enrichment of the DEGs from spleen tissue between WZS pigs and LW pigs; Table S11: The KEGG enrichment of the DEGs from LDM tissue between WZS pigs and LW pigs; Table S12: The gene expression of 12 genes in different tissues; Table S13: The 2^−ΔΔCT^ values of the significantly different expression genes of RT-qPCR results.
