# Supplementary material for: The Genetic Selection of HSPD1 and HSPE1 Reduce Inflammation of Liver and Spleen While Restraining the Growth and Development of Skeletal Muscle in Wuzhishan Pigs
Source: Animals (Basel). 2024 Jan 4;14(1):174. doi: 10.3390/ani14010174 (PMC10777996; doi:10.3390/ani14010174)
Supplement: Supplementary file 1 [file animals-14-00174-s001.zip › Supplementary figures.pdf]

Supplementary Figures

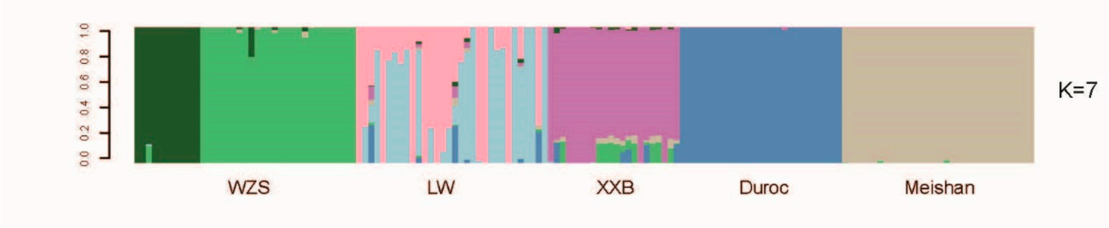

Figure S1. The optimal number K=7 of the population structure

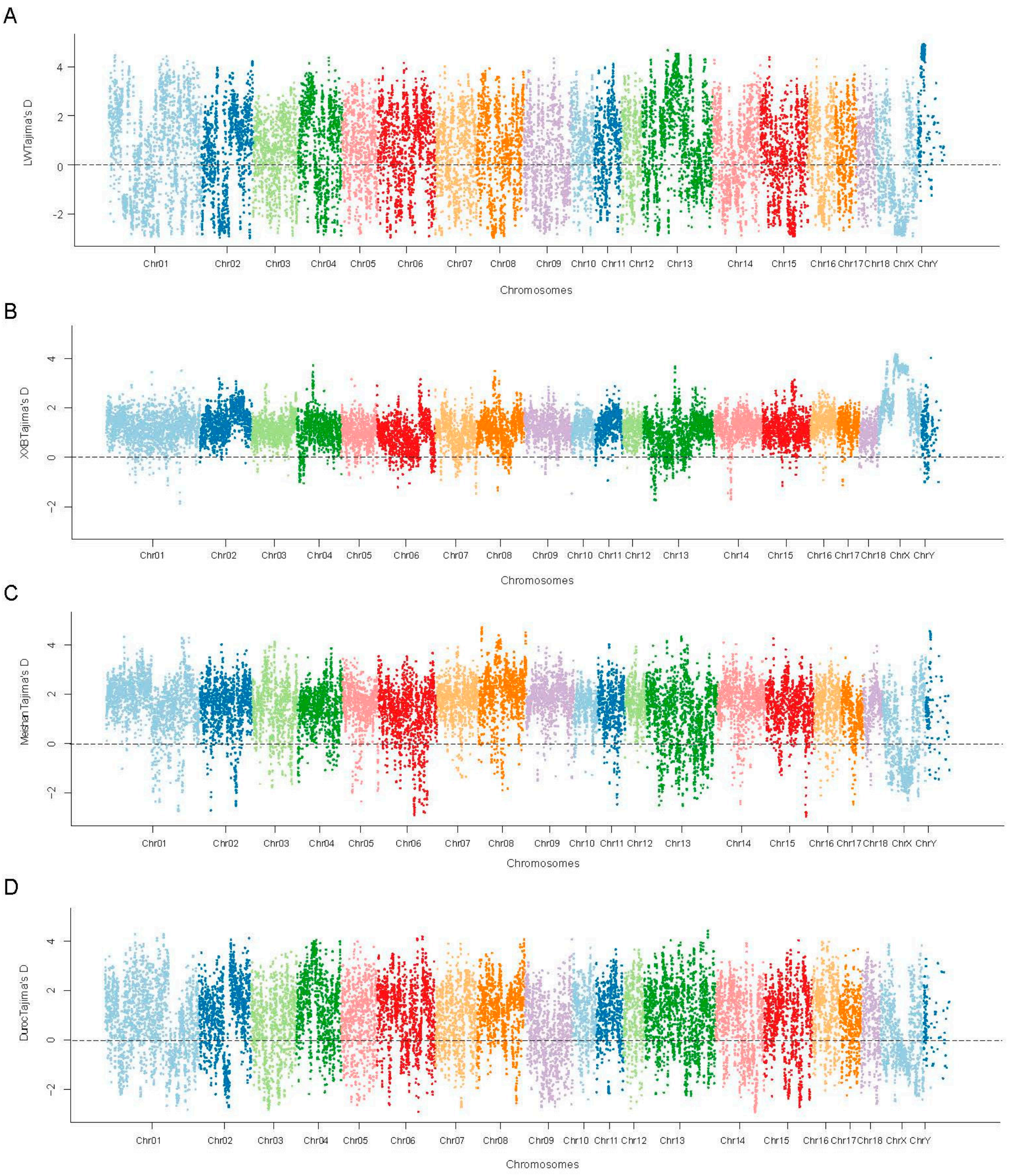

Figure S2. The Tajima's D distribution on the chromosomes of four pig populations. **(A)** LW. **(B)** XXB. **(C)** Meishan. **(D)** Duroc.

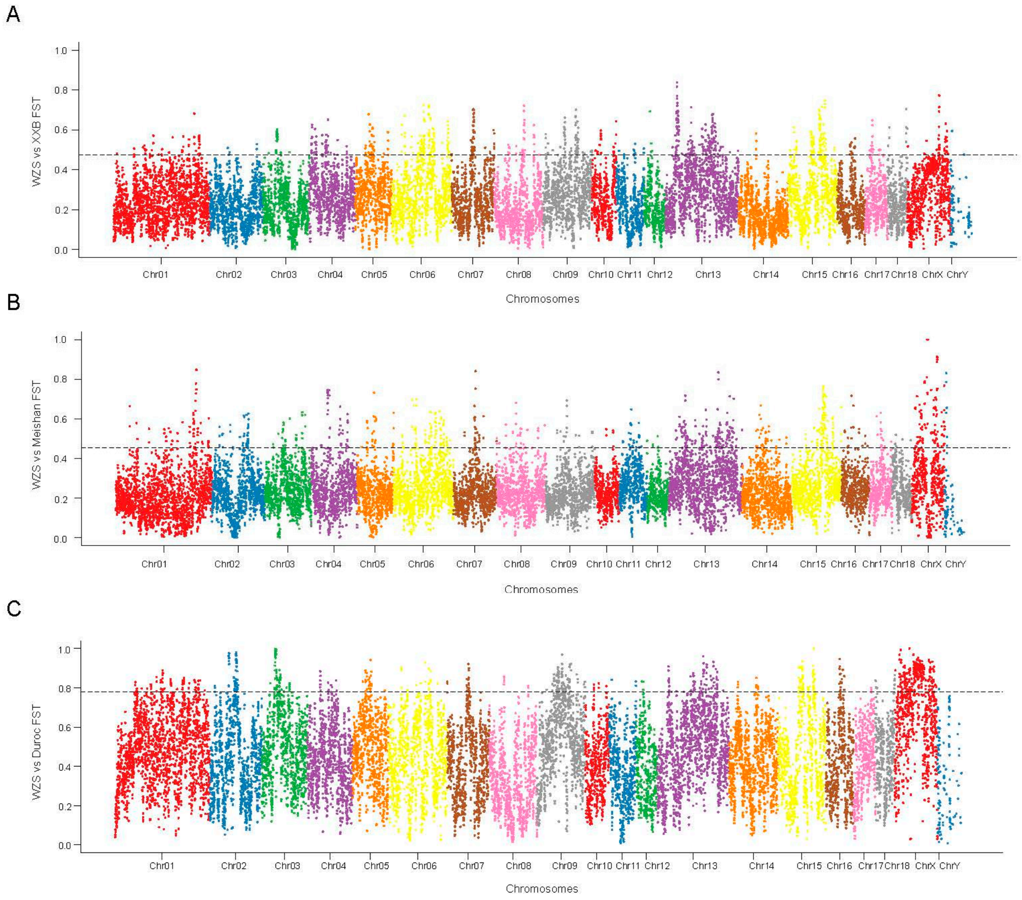

Figure S3. The FST distribution on the chromosomes between WZS pigs and other pigs. **(A)** WZS and XXB. **(B)** WZS and Meishan. **(C)** WZS and Duroc.

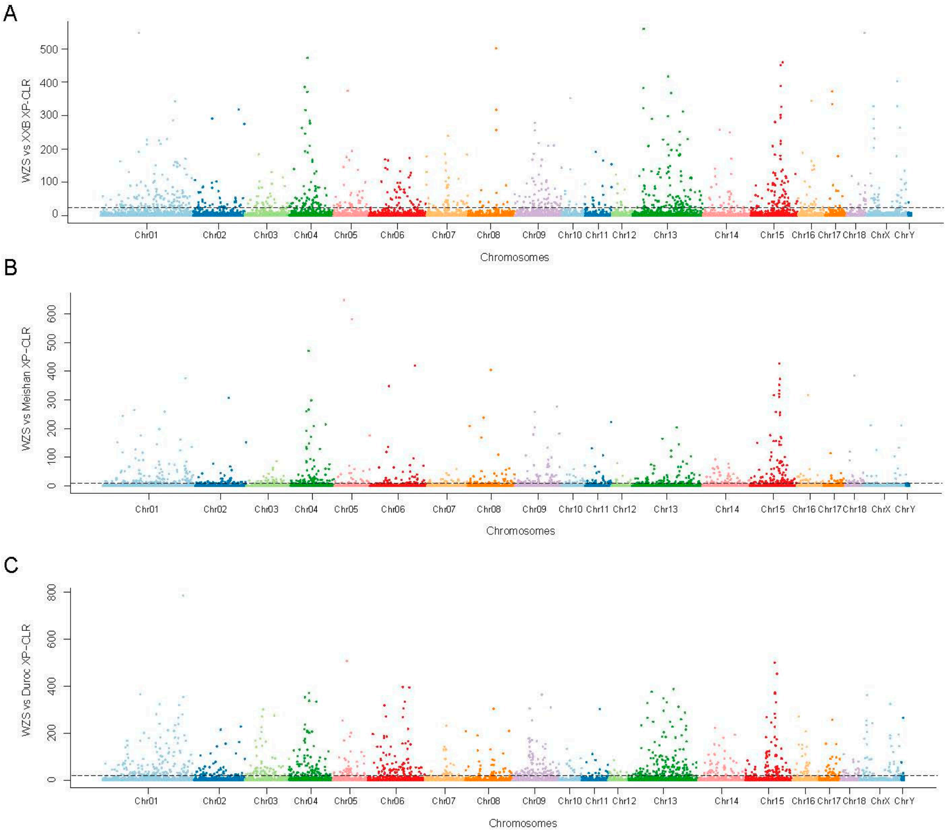

Figure S4. The XP-CLR distribution on the chromosomes between WZS pigs and other pigs. **(A)** WZS and XXB. **(B)** WZS and Meishan. **(C)** WZS and Duroc.

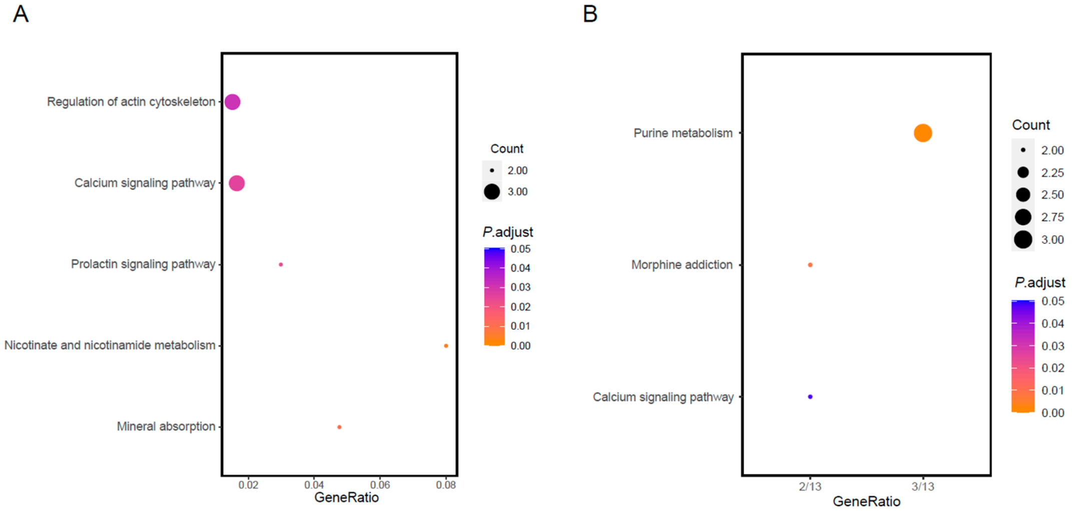

Figure S5. The KEGG enrichment of the top 5% genetic area between WZS and other pigs. **(A)** WZS and Meishan. **(B)** WZS and Duroc.
